# Tracking temporal progression of benign bone tumors through X-ray based detection and segmentation

**DOI:** 10.1038/s41598-025-23053-4

**Published:** 2025-11-11

**Authors:** Se-Yeol Rhyou, Chohee Bang, Yong Jin Cho, Hyunjae Bae, Yun Ju Ha, So-Young Baek, Yeonhu Lee, Choonok Kim, Jeong Eun Moon

**Affiliations:** 1 Department of Electrical and Computer Engineering, College of Information and Communication Engineering, Sungkyunkwan University, Suwon, 440-746 South Korea; 2 Cleverus Corp, Seoul, 06771 Republic of Korea; 3 Department of Nursing, Honam University, 100 Honamdaegil, Gwangsangu, Gwangju, 62399 Republic of Korea; 4 Department of Orthopedic Surgery, Chosun University College of Medicine, Gwangju, 61453 Republic of Korea; 5 J INTS BIO Inc, Seoul, Republic of Korea

**Keywords:** X-ray, Benign bone tumor, Object detection, Semantic segmentation, OCR, Medical imaging, Bone imaging

## Abstract

X-ray is the most widely used imaging modality for the initial diagnosis of bone tumors due to its accessibility and cost-effectiveness. However, the longitudinal comparison of benign bone tumors, particularly for assessing size and shape progression over time, remains largely manual and subjective. In this study, we propose FusionX-BBTNet, a deep learning-based framework that enables automated detection, segmentation, and time-sequential analysis of BBTs from X-ray images. The framework combines YOLO-based object detection with U-Net segmentation, and utilizes a novel wavelet-enhanced dataset to improve contour accuracy. To enable real-world quantification, an OCR-based module is used to extract the X-ray scale bar and compute the pixel-to-length conversion ratio. With this, tumor size and area are calculated in millimeters, and their changes over time are visualized through centroid-based alignment. The proposed method was validated on a dataset of 466 expert-annotated X-ray images, achieving a mean IoU of 0.9376 and a boundary F1 score of 0.9827. In addition to providing reliable tumor localization and measurement, the system supports clinical decision-making by offering intuitive shape and area comparisons. This approach has the potential to complement expert interpretation and improve diagnostic efficiency, especially in environments with limited radiological expertise.

## Introduction

In the diagnosis of bone tumors, plain radiography (X-ray) is the most basic and important imaging test^1,2^. X-rays are a diagnostic method that is very familiar to doctors including orthopedic surgeons, and since they are relatively inexpensive and can be taken according to standard protocols at any hospital, a certain level of quality is guaranteed^3^. In X-ray, the anatomical location of the lesion, the zone of transition between the lesion and the host bone, and features within the lesion are helpful in determining the diagnosis and extent of the disease^4^. When the zone of transition between the host bone and the lesion is wide and the border is unclear, the term permeative pattern is used, suggesting that the lesion has aggressive characteristics^5,6^. This phenomenon occurs because the disease progresses quickly, leaving insufficient time for the host bone to react^7^. Conversely, a narrow zone of transition with clear boundaries suggests a benign bone tumor (BBT)^8^. Lodwick et al. described the form of bone destruction as geographic, moth-eaten, and permeative, reflecting the rate of progression of the lesion, depending on the rate of tumor growth^9^. If a bone tumor destroys cortical bone or exhibits periosteal reactive osteogenesis, it should be considered an aggressive tumor^10,11^. Occasionally, the cortical bone is not destroyed, but instead swells and thins, which is mainly seen in BBTs such as aneurysmal bone cysts, fibrous dysplasia, and enchondromas^12^. If the X-ray findings suggest a benign bone tumor, serial X-ray follow-up is important in establishing a diagnosis and patient treatment plan^13^.

In the diagnosis of bone tumors, comparing X-rays of the same area at different times can help determine changes in size, shape, and accompanying findings, including calcification, and it is very important for diagnosis^14–16^. It is also very helpful in deciding whether to perform further evaluations, such as MRI with enhancement or whole-body bone scan^17,18^. Until now, when comparing X-rays of the same part at different times in bone tumor cases, different X-rays were displayed on the same screen and compared by eye, and there were no other digitalized or quantified tools^19–21^. Therefore, each doctor made different decisions based on their own characteristics. To overcome these limitations, various AI-powered solutions have been proposed in recent years, focusing on the automatic detection, segmentation, and temporal monitoring of BBT^22^.

Von Schacky et al.^20^ introduced a multitask deep learning model capable of both segmenting and classifying primary bone tumors on plain radiographs. The model was trained on 1,356 radiographs with histopathologically confirmed labels, achieving high performance in tumor classification and generating segmentation masks that delineate tumor boundaries. This approach is notable in that it moves beyond simple detection and enables spatially-aware tumor interpretation. However, the study did not quantify tumor size or surface area, nor did it address time-series comparisons. In addition, while segmentation masks were generated, these were not utilized to extract clinically meaningful metrics such as length, width, or area, limiting its applicability in longitudinal tracking or treatment monitoring.

ŞIMŞEK et al.^22^ proposed a deep learning model for the automatic detection of enchondroma on X-ray images by modifying the Detectron2 architecture. Trained on pathologically confirmed data, the model achieved a high accuracy of 0.9899, demonstrating strong potential for early clinical diagnosis of benign bone tumors (BBTs). However, the study is limited to single-timepoint analysis and does not provide quantitative measurements such as tumor area or length. It also lacks the ability to track temporal changes and is restricted to enchondroma, limiting its generalizability across various BBT types.

The authors aimed to develop a reliable AI module to compare X-rays of the same bone tumor site at different time points. In this study, we propose FusionX-BBTNet, an automated framework for the detection, segmentation, and time-sequential comparison of BBTs in X-ray images. The system combines YOLO^23^-based object detection and U-Net^24^ segmentation with a novel wavelet-enhanced dataset to improve accuracy in contour extraction. By applying Optical Character Recognition (OCR)^25,26^-based pixel-to-length conversion, the model enables precise measurement of tumor size and area in real-world units. In addition, a temporal alignment technique is introduced to support longitudinal shape comparison. The proposed framework offers a reliable and interpretable tool for clinicians, particularly in settings with limited radiological expertise, and demonstrates strong potential as a clinical decision support system.

## Methods

### Dataset preparation

We collected anonymized X-ray images from patients at Chosun University (CSU) Hospital who underwent radiographic evaluation under diagnostic codes D160 (benign neoplasm of long bones of upper limb), D161 (short bones of upper limb), D162 (long bones of lower limb), and D163 (short bones of lower limb). The study was approved by the Institutional Review Board of Chosun University Hospital (IRB No. CHOSUN-2025-04-008), and informed consent was obtained from all subjects and/or their legal guardian(s) prior to participation. The data were randomly selected regardless of age or sex. All images were of uniform resolution (1125 × 1125 pixels), acquired using a digital X-ray system (GC85A, Samsung Electronics, Suwon, South Korea) at a fixed source-to-image distance of 200 cm. The ground-truth measurements for tumor size and area were manually annotated by musculoskeletal radiologists at CSU Hospital. In total, the dataset consisted of 466 X-ray images, which were used for both object detection and semantic segmentation. Of these, 316 images were used for training, 100 for validation, and 50 for testing. The dataset was divided on a patient-wise basis to ensure that no subject appeared in both training and test sets. In addition, 5-fold cross-validation was conducted to enhance robustness and prevent overfitting.

### Detecting BBT and edge information

In this section, we aim to detect BBT, measure their horizontal and vertical lengths, and analyze their shapes. To achieve this, we first apply an object detection method to localize the BBT within the X-ray image using bounding boxes. Within these regions, semantic segmentation is performed to precisely delineate the BBT area. Subsequently, we apply wavelet transform^27^ to the segmented regions to extract the LL, LH, and HL components, while treating the HH component as noise. Rather than reconstructing the image using conventional methods, we repurpose the wavelet components into a novel three-channel format: LL is assigned to the red channel, LH to green, and HL to blue. This results in a new 3-channel image representation, referred to as the 3-Channel Reconstructed BBT (FusionX-BBT). We then concatenate this image with the original grayscale X-ray to form a 4-dimensional dataset, which enables more precise localization of BBTs. By recognizing the scale bar present in the X-ray, we compute the real-world length per pixel, allowing us to estimate the actual size of the tumor. In addition, edge extraction is applied to analyze shape variations of the tumor across sequential time-series data. The following subsections provide a detailed explanation of each step in the process.

#### BBT detection

First, to detect BBTs from anonymized X-ray images, we initially applied an object detection model to localize the tumor regions. This step allowed us to estimate the approximate location of the BBT, which significantly reduced the computational burden in the following process. Based on the detected region, a semantic segmentation technique was applied to accurately delineate the tumor contours.

**Fig. 1:**
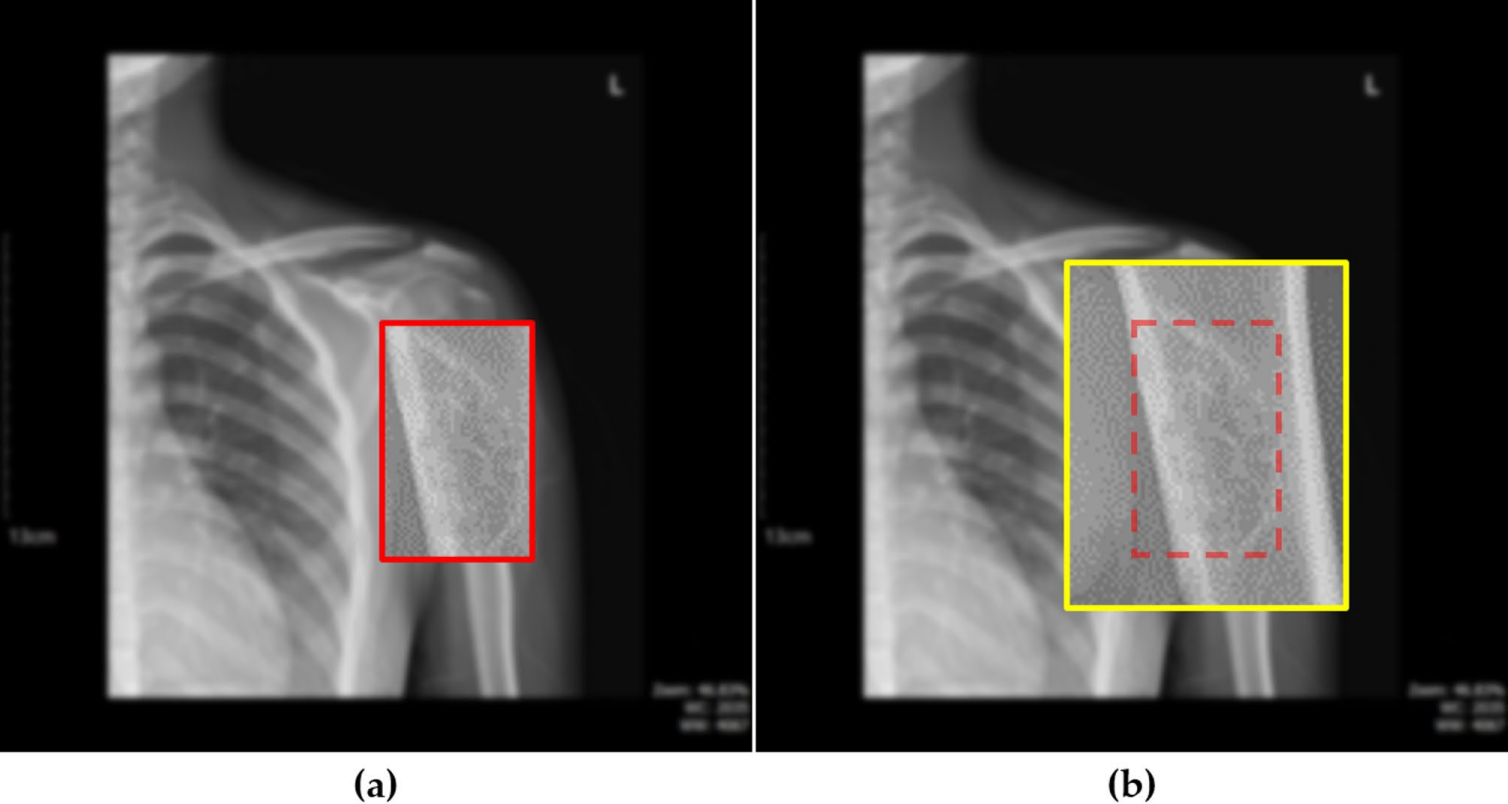
(**a**) Magnified image showing a detected BBT located in the humerus. (**b**) The red dashed box corresponds to the bounding box shown in image (a), while the yellow box represents the same region with an additional 20-pixel padding applied to all sides.

To determine the most suitable detection approach, we compared the performance of three widely used models in the YOLO family: YOLOv5, YOLOv8, and YOLOv11. The evaluation was conducted using the mean Average Precision at an IoU threshold of 0.5 (mAP@0.5)^28^ as the primary metric. Each model was briefly reviewed in terms of its architectural characteristics and its strengths and weaknesses in the context of medical imaging. YOLOv5 is a lightweight, PyTorch-based model that offers rapid inference and flexible architectural scaling, making it advantageous for real-time diagnostic support. YOLOv8 enhances detection accuracy with an improved backbone and refined feature extraction, allowing for more precise localization of small tumors in a variety of medical imaging environments. YOLOv11, the most advanced version, incorporates a transformer-based structure along with a highly sophisticated feature extraction network, enabling robust performance on complex radiographic data. We conducted a comparative analysis using real medical image datasets to assess the detection performance of each model and to identify the most appropriate one for BBT detection. To ensure that the tumor boundaries were fully retained, additional padding was applied to all four sides of the detected bounding boxes, as illustrated in Fig. 1.

#### BBT preprocessing

To achieve precise segmentation of BBTs, we first localized the tumor region within the X-ray image using a YOLO-based object detection model. Since one of the ultimate objectives of this study is to analyze the morphological changes of tumors, it was critical to preserve contour information. Therefore, we added a fixed padding around the detected bounding box to minimize the risk of losing boundary details. Once the padded bounding box was generated, we applied a preprocessing step. Although X-ray images generally exhibit high clarity, they inevitably contain a certain level of noise due to the inherent characteristics of medical imaging. To address this, we employed wavelet transform, a widely used noise reduction technique. Wavelet transform is a mathematical approach that decomposes a signal or image into its frequency components, making it particularly effective for multi-resolution analysis. This allows us to separate low-frequency components, which represent overall structure and shape, from high-frequency components, which typically include noise and fine details such as edges. The strength of wavelet transform in medical imaging lies in its ability to reduce high-frequency noise while preserving essential anatomical structures and contours. In clinical images, suppressing background noise without distorting the tumor shape is crucial, and wavelet transform is well-suited to this purpose. In our study, we applied a Haar-based wavelet transform to the tumor-containing regions within the padded bounding box. This enabled us to reduce noise while retaining the contour and shape of the BBT, thereby facilitating more accurate results in the subsequent segmentation process.

**Fig. 2:**
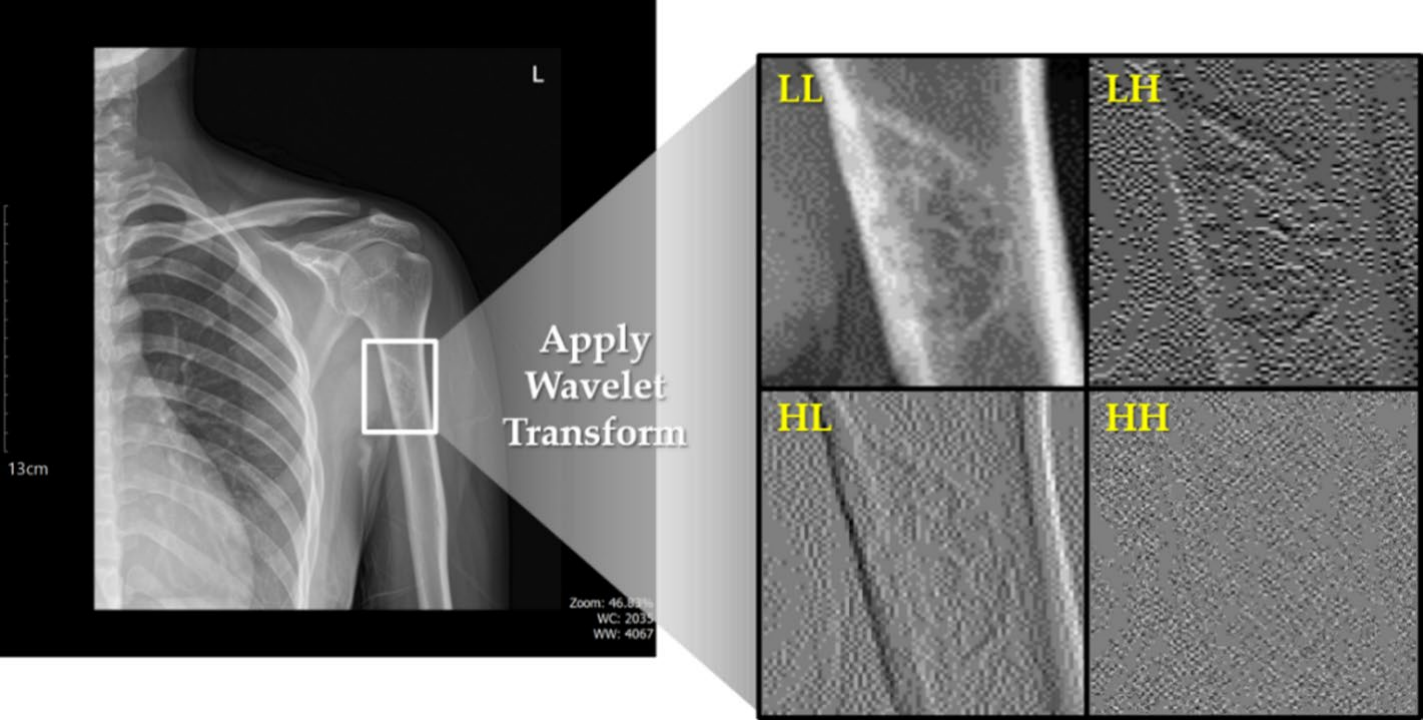
Result of applying wavelet transform to a BBT located in the humerus of a patient.

By applying wavelet transform to the padded bounding box, we obtained results as shown in Fig. 2. Among the resulting components, the HH component was considered as noise and thus excluded from further processing. Using the remaining LL, LH, and HL components, we generated a new dataset referred to as the FusionX-BBT dataset. To create this dataset, we restructured the original single-channel image into a three-channel format. Specifically, the LL component was assigned to the red channel, the LH component to the green channel, and the HL component to the blue channel. Following this, the newly constructed three-channel image was concatenated with the original grayscale image, resulting in a four-channel representation of the data.

**Fig. 3:**
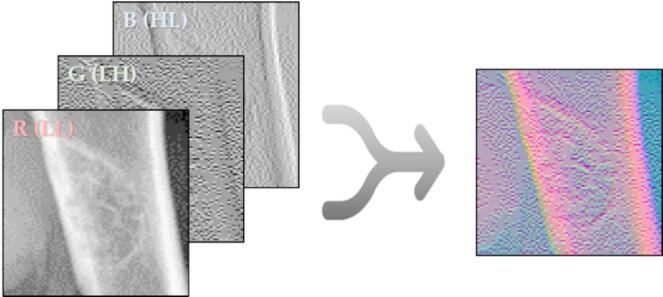
FusionX-BBT dataset generated with LL, LH, HL component.

This approach represents a clear departure from conventional wavelet-based denoising or simple reconstruction techniques. Traditional wavelet reconstruction typically emphasizes the LL component while treating the high-frequency components such as HL, LH, and HH as noise to be either suppressed or discarded. Although effective in noise reduction, this process often leads to the unintended loss of fine structural and edge details. In this study, we took a different direction by mapping the LL, LH, and HL components to the red, green, and blue channels of a three-channel image. This strategy was designed to overcome the limitations of single-channel representations by capturing a broader spectrum of features. The LL component contains information on overall structure and shape, whereas the LH and HL components capture edge features along the vertical and horizontal axes, respectively. As illustrated in Fig. 3, allocating each component to a separate RGB channel brings together various types of features such as shape, texture, and boundary within a single-color image. Through this method, the resulting image contains a significantly richer set of features compared to a conventional grayscale image. When used as input to a deep learning-based segmentation model, this image enables the model to learn more diverse and informative representations. Additionally, key morphological and structural characteristics, which are essential in medical imaging, can be more effectively preserved. By concatenating this enhanced image with the original grayscale version, we create a data representation that offers a more complete and informative input for subsequent analysis.

#### BBT segmentation

**Fig. 4:**
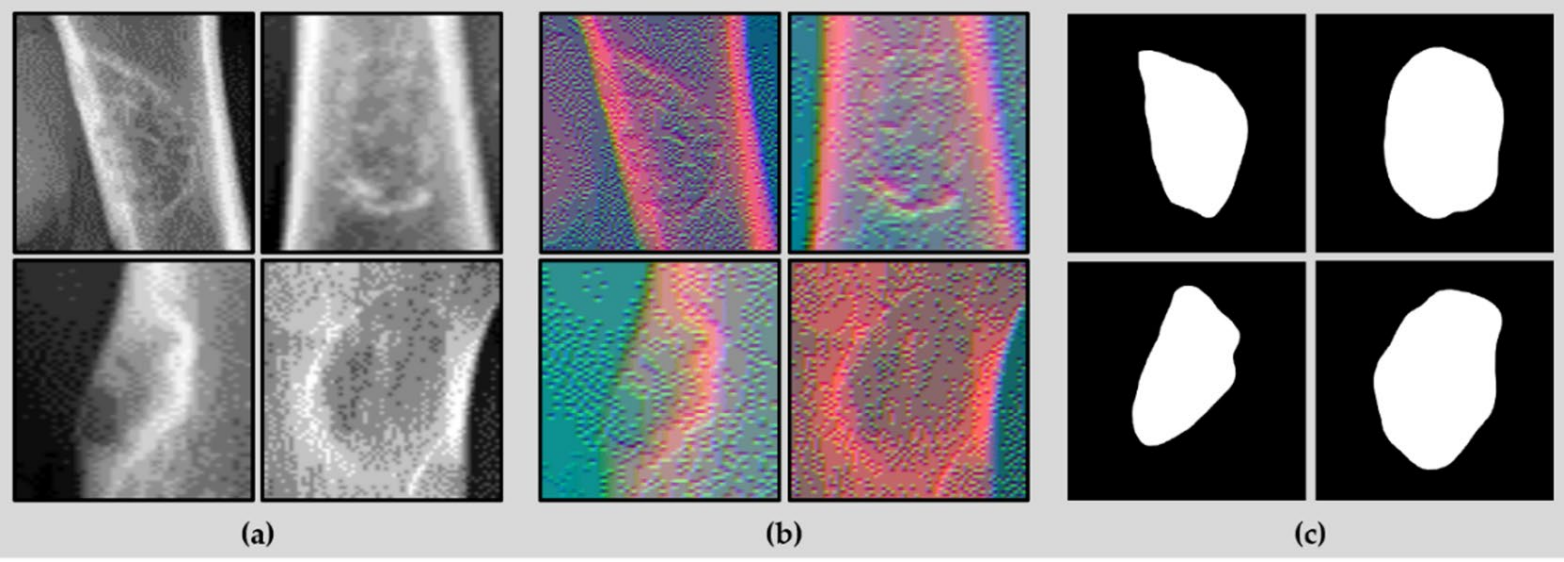
(**a**) BBT region cropped image. (**b**) FusionX-BBT dataset (**c**) Label of dataset.

Using the FusionX-BBT dataset, we performed semantic segmentation to accurately determine the location, size, and shape of the BBT. To validate the effectiveness of our newly constructed dataset, we conducted a comparative analysis across three different data configurations. These included a dataset cropped from the BBT region in the raw grayscale image, the FusionX-BBT dataset itself, and a four-dimensional dataset formed by concatenating the raw image and FusionX-BBT image. As illustrated in Fig. 4(a) and 4(b), each dataset contains the same tumor region, allowing the use of a common ground truth label. For the segmentation model, we selected U-Net, a widely adopted architecture in the field of medical imaging. Figure 4(c) shows the resulting output of the process. Although more advanced transformer-based networks such as TransUNet^29^ or Swin-Unet^30^ could also have been employed, the primary objective of this study was to evaluate the utility of the proposed dataset. Given this goal, U-Net was considered more appropriate due to its relatively simple structure and fast training capability.

#### BBT edge detection

In the next stage, we aimed to extract the edge of the segmented BBT. To clearly visualize the contour of the labeled BBT region, we applied a contour detection-based method. Unlike traditional gradient-based edge detection techniques such as Canny^31^, our approach directly extracts the contour from a binary label mask. This allows for the generation of sharper and more continuous contours. Given a label mask $\:L\left(x,y\right)\in\:\{0,\:1\}$, where the value of 1 represents the BBT and 0 denotes the background, the set of edge pixels *E* is defined as follows:

1 $$\:E=\left\{\left(x,y\right)\:\right|\:L\left(x,y\right)=1\:\wedge\:\exists\:\left({x}^{{\prime\:}},\:{y}^{{\prime\:}}\right)\in\:\mathcal{N}\left(x,\:y\right),\:L\left({x}^{{\prime\:}},\:{y}^{{\prime\:}}\right)=\:0\}\:$$

where $\:\mathcal{N}\left(x,\:y\right)$ denotes the eight-connected neighborhood of the pixel *(x*,* y)*, representing the logical condition used to detect edge pixels located at the boundary between the labeled tumor and the background. This equation allows for precise edge detection based on the shape of the object, ensuring that only the contour is extracted without including the internal area of the label. Such a method contributes to improved visual interpretation accuracy in image-based disease diagnosis and analysis by providing a clear and continuous representation of anatomical boundaries.

### Detecting scale bar

To quantitatively measure the actual size of the BBT, we utilized the scale bar embedded within the X-ray image. The scale bar contains spatial reference information that indicates the real-world distance per pixel, serving as a critical basis for converting image-based measurements into millimeter units. In this study, we isolated the leftmost 10% of the X-ray image for analysis. By focusing on this region independently of the main anatomical area, we were able to simplify the visual conditions and extract the characteristics of the scale bar more effectively.

**Fig. 5:**
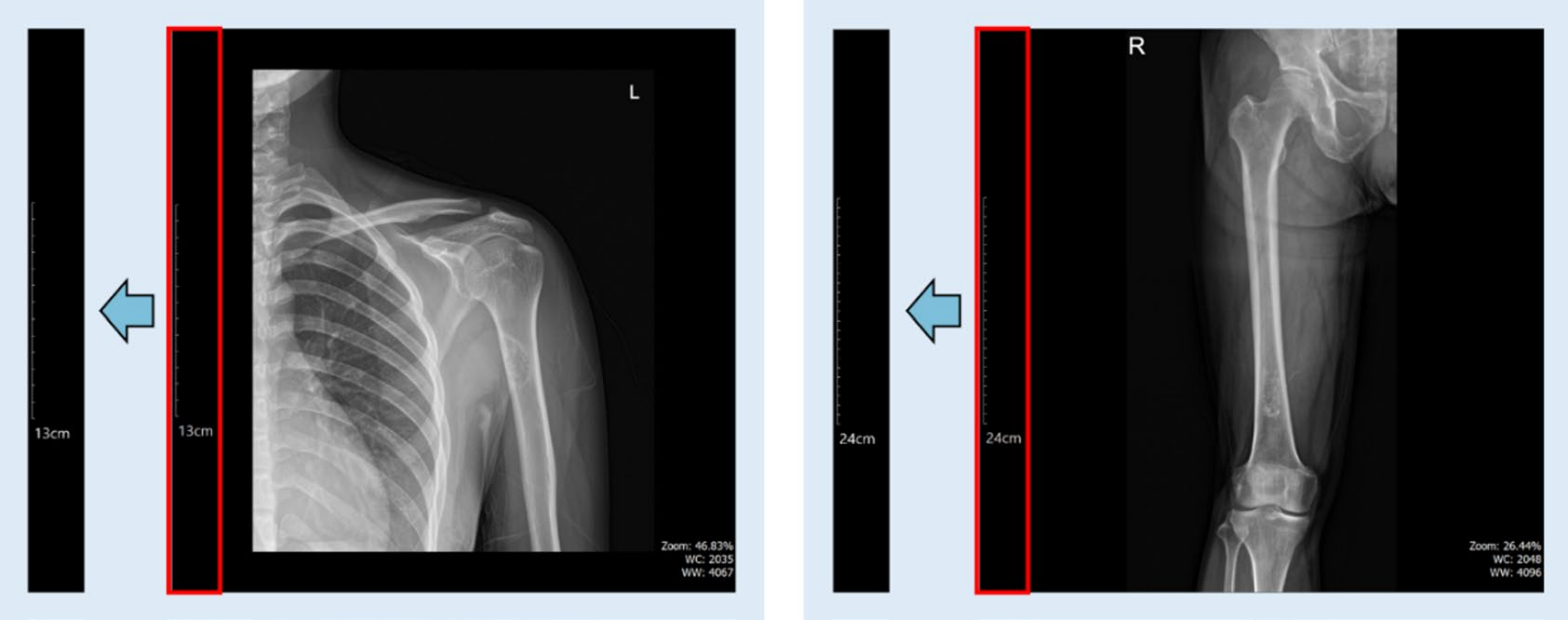
Extracted view of the scale bar region.

The extracted region consisted of a grayscale image; however, in some cases, the boundaries of the scale bar were unclear due to image blurring or low contrast. To address this, a binarization process was applied in which pixel values less than or equal to 25 were set to 0, and values greater than 25 were set to 255. Following this, the pixels with a value of 255 were grouped into connected components, and the component with the greatest vertical height was selected. The height of this component corresponds to the actual pixel length of the scale bar and serves as a reference for calculating the conversion ratio.

In order to accurately convert pixel measurements into real-world distances, it was necessary to automatically recognize the numeric value indicated on the scale bar. For this purpose, we applied OCR. In this study, we used EasyOCR^32^, a pre-trained deep learning-based OCR model known for its high recognition accuracy in medical imaging, even without additional training. EasyOCR is based on a Convolutional Recurrent Neural Network (CRNN) architecture that integrates Convolutional Neural Networks (CNN) and Recurrent Neural Networks (RNN), and employs an attention-based decoder to effectively detect and interpret characters and digits within images. One of its key advantages lies in its robustness under challenging conditions, such as poor contrast and the presence of noise, which are common in clinical imaging.

As illustrated in Fig. 5, OCR was performed on the preprocessed region of interest (ROI), typically recognizing strings such as “10 cm” or “24 cm.” The recognized text was then post-processed to separate the numeric value and the unit. The value in centimeters was converted to millimeters and, along with the previously measured pixel height, was used to compute the conversion ratio representing millimeters per pixel. This information was visually overlaid as yellow text at the top of the image, while the recognized text region and the corresponding scale bar location were highlighted with cyan bounding boxes. This visualization enhanced both the interpretability and reliability of the results. Through this method, accurate spatial information was efficiently extracted from X-ray images without the need for additional training. This allowed for reliable calculation of the actual tumor size and area, thereby improving the overall precision of subsequent quantitative analyses.

### Calculate BBT size and area

Using the pixel-to-millimeter conversion ratio obtained from the X-ray image, we quantitatively calculated the actual size and area of the BBT. Accurate measurement of tumor size based on medical imaging plays a critical role in assessing disease progression and serves as a fundamental preprocessing step for planning clinical interventions such as surgery or radiotherapy. In this study, all size and area measurements were converted into real-world units (millimeters) based on the distance per pixel automatically estimated through OCR. This enabled consistent and reliable quantification of tumor dimensions across different images.

**Fig. 6:**
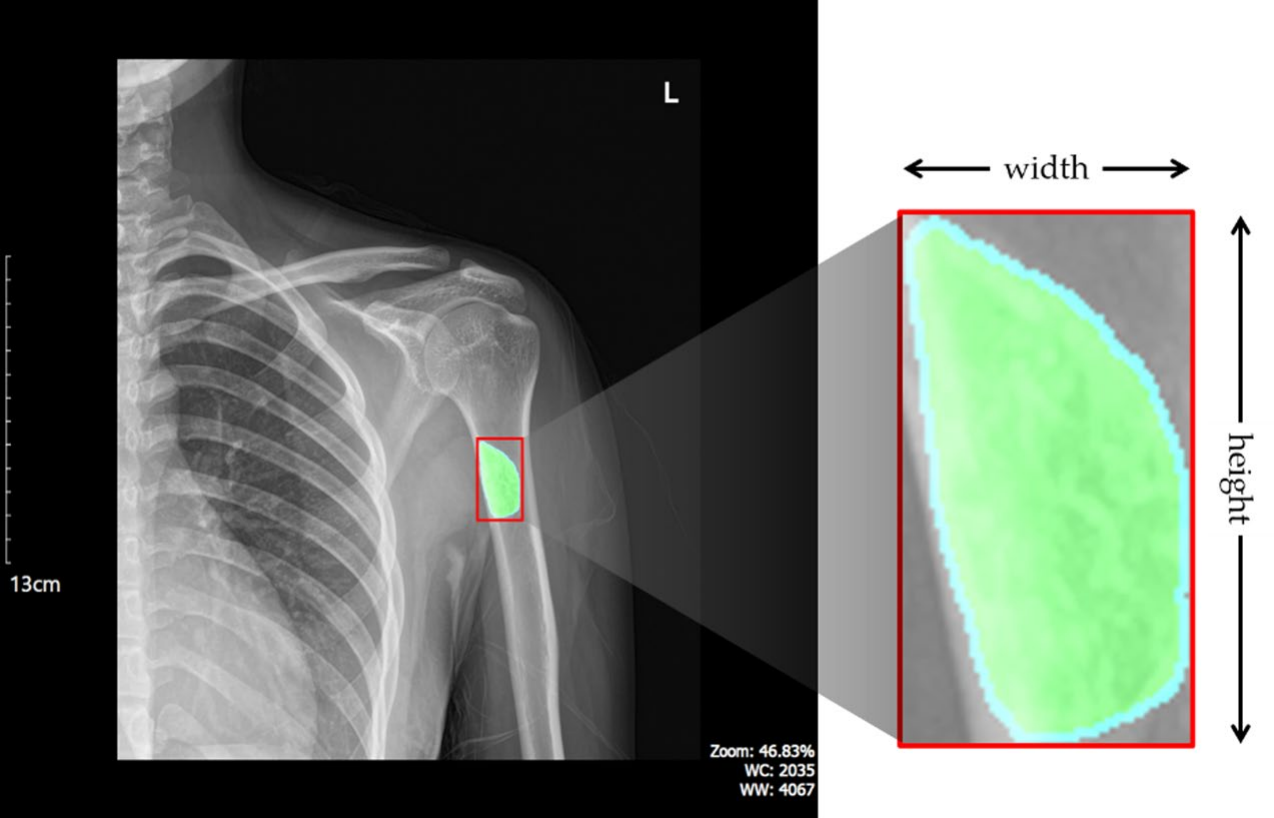
Width and height of minimum bounding box created by BBT region.

As illustrated in Fig. 6, the size of the BBT was measured using the minimum bounding box that enclosed the tumor region. The width and height of the bounding box were first calculated in terms of pixel length. These values were then converted to real-world distances in millimeters by applying the pixel-to-millimeter conversion ratio obtained through OCR. Let *W*_*pixels*_ and *H*_*pixels*_ denote the width and height of the bounding box in pixels, and let *S*_*mm/pixel*_ represent the spatial resolution in millimeters per pixel. The actual width and height of the BBT can be expressed as follows:

2 $$\begin{aligned}\:Width\left(mm\right)={W}_{pixels}\times\:{S}_{mm/pixel} \\ \:Height\left(mm\right)=\:{H}_{pixels}\times\:\:{S}_{mm/pixel}\end{aligned}$$

The actual tumor length, once calculated, was visually presented on the result image using bidirectional arrows and numerical values, providing an intuitive understanding of the measurements. Next, the area of the BBT was calculated by summing all pixels corresponding to the tumor region in the label image. Let *N*_*pixels*_​ represent the total number of tumor pixels. The actual area of the BBT in the X-ray image, denoted as *BBT*_*area*_​, was computed as follows:

3 $$\:{BBT}_{area}=\:{N}_{pixels}\times\:{\left({S}_{mm/pixel}\right)}^{2}$$

In other words, the actual area was calculated by multiplying the number of tumor pixels by the physical area represented by a single pixel. This approach directly reflects the real-world spatial coverage of each pixel, allowing for precise measurement of the BBT’s irregular and complex shape. It also prevents the common problem of area overestimation seen in bounding box-based calculations and minimizes the loss of morphological detail that often occurs with simplified models such as elliptical approximations. To enhance visual interpretability, the tumor boundary was extracted using an edge detection method and overlaid on the result image as a yellow contour. This enabled a direct comparison between the calculated area and the corresponding bounding box, allowing users to verify their consistency at a glance. The tumor contour, bidirectional arrows for length measurement, and the area value were all displayed simultaneously within the same frame, contributing to both the reliability and interpretability of the analysis. This method provides an efficient and accurate means of estimating the actual size and area of BBTs in X-ray images without the need for additional training processes. As such, it serves as a practical and quantitative tool to support clinical diagnosis and treatment planning.

### Compare sequential BBT dataset

We have now completed the preparatory procedures necessary for conducting a time-sequential comparison of BBT size and shape, which represents the ultimate objective of this study. For this purpose, time-series X-ray images were collected from a single patient at CSU Hospital. The images were acquired at intervals ranging from six months to one year. Given the nature of BBTs, there should be no significant change in tumor size or morphology over time under normal circumstances. However, if variations exceeding a certain threshold are observed, it may suggest the potential progression of the lesion. This may require additional imaging, biopsy, or adjustment of the treatment strategy. In this study, we defined a quantitative criterion for clinical significance. Specifically, a change in BBT area greater than 10%, whether an increase or decrease, was considered meaningful. Changes in contour shape were not evaluated based on fixed criteria, as such assessments are more appropriately made by clinical experts. Instead, shape changes were used in a supportive role to aid interpretation. This approach enabled both visual and quantitative comparison of tumor progression over time, providing a foundation for more objective evaluation in clinical settings.

**Fig. 7:**
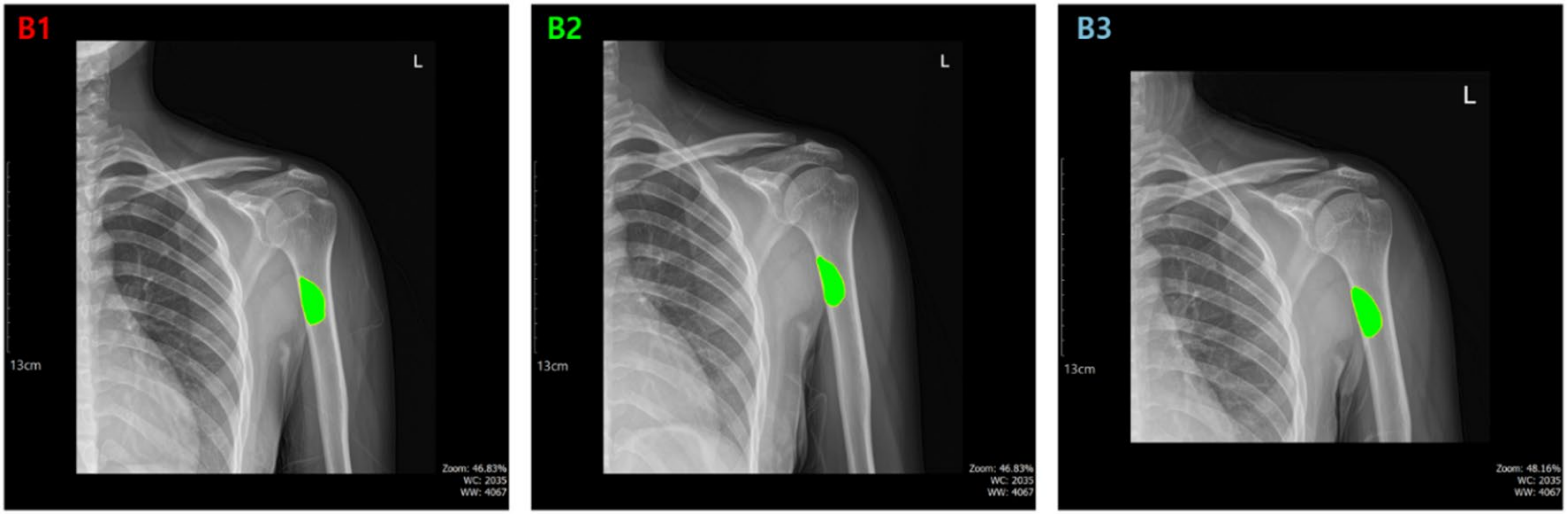
Sequential X-ray images of the humerus from a single patient, taken over a one-year period, with segmented regions corresponding to BBT areas.

To conduct this analysis, we segmented the BBT regions from multiple time points obtained from a single patient and visualized them within a single frame for comparison. Figure 7 shows X-ray images of a patient with a BBT in the humerus, along with the segmentation results for three images captured approximately one year apart. Each BBT region extracted at different time points was separately cropped and arranged side by side, allowing for intuitive comparison of changes in size and shape over time.

**Fig. 8:**
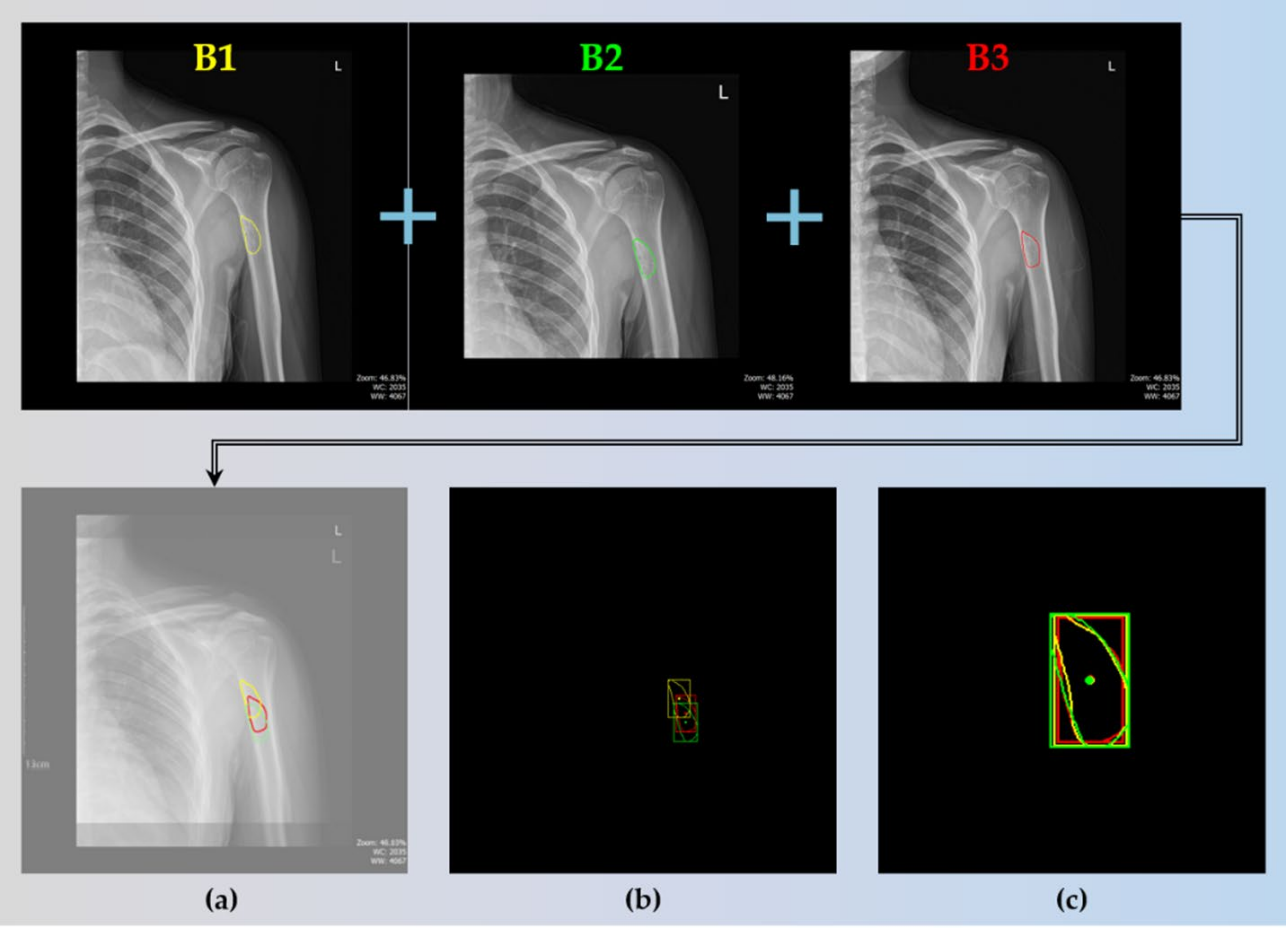
X-ray images B1, B2, and B3 acquired at one-year intervals. (**a**) Overlaid image combining B1, B2, and B3 to visualize changes over time. (**b**) Cropped edges of the BBT regions with corresponding bounding boxes and computed centroids. (**c**) BBT regions realigned based on their centroids to assess shape variations across time points.

To achieve this, we first combined the images B1, B2, and B3, each taken at one-year intervals, as shown in Fig. 8. We then extracted only the segmented BBT regions. As illustrated in Fig. 8(b), the extracted tumor regions were initially distributed in different positions. To align them, we generated a bounding box for each BBT and calculated its centroid. Using the centroid as a reference point, we repositioned the tumors to a common location. This alignment allowed us to observe changes in BBT shape over time, as demonstrated in Fig. 8(c).

In light of the growing shortage of radiology specialists, the proposed method offers a valuable tool for non-specialists and clinicians to intuitively assess the presence or absence of changes in BBTs. By enabling rapid clinical decision-making and facilitating timely treatment planning, this approach holds significant potential for improving both the clinical utility and overall efficiency of patient care.

## Result

The proposed CSM-FusionNet was implemented using MATLAB R2023b on a workstation equipped with an NVIDIA GeForce RTX 3090 GPU with 24 GB of memory. Expert-annotated BBT X-ray images were obtained from CSU Hospital to evaluate the performance of the model. As demonstrated in the experimental results, FusionX-BBTNet achieved significant performance improvements in the detection and segmentation of BBTs. The process of constructing the FusionX-BBT dataset involved applying wavelet transform to effectively suppress noise while enhancing tumor contours. By combining the transformed image with the original grayscale image, a four-channel input was created, providing a richer set of features and improving segmentation performance. Accurate segmentation directly contributed to more precise measurements of BBT size and area. Furthermore, enhanced edge detection enabled the model to capture shape variations with near-perfect accuracy, facilitating robust analysis of morphological changes over time.

### Performance of BBT detection

To evaluate the performance of BBT detection, we conducted comparative experiments using three widely adopted models from the YOLO family. Specifically, the detection performance of YOLOv5, YOLOv8, and YOLOv11 was assessed based on two criteria: the mean Average Precision at an mAP@0.5 and the actual detection success rate on the test dataset. Details on the rationale for selecting these three models can be found in the BBT detection section. This comparison was designed to identify the most effective model architecture for BBT localization in clinical X-ray images, as summarized in Table 1.

**Table 1:** The mAP@0.5 scores for each network and the detection results on the test data.

Network	mAP@0.5	Test Accuracy
YOLOv5	0.994	0.98
YOLOv8	0.987	0.94
YOLOv11	0.982	0.92

Interestingly, YOLOv5 demonstrated superior performance in BBT detection compared to YOLOv11, the most recent model in the YOLO series. To ensure a fair comparison, YOLOv5, YOLOv8, and YOLOv11 were all evaluated under identical experimental conditions. The input image size was set to 736 by 736 pixels, training was performed for 100 epochs, and the batch size was fixed at 16. Among the three models, YOLOv5 achieved the highest mAP at an IoU threshold of 0.5 and also recorded the highest detection accuracy on the test dataset.

These results indicate that a more recent model does not necessarily guarantee better performance. YOLOv5 has undergone extensive optimization over time in various practical environments. Its relatively simple and lightweight architecture allows it to generalize well in specialized scenarios such as medical imaging, where data is often limited and lesions are typically small in size. A key architectural difference lies in the use of anchors. YOLOv5 employs an anchor-based approach, while YOLOv8 and YOLOv11 adopt anchor-free mechanisms. Although anchor-free designs offer greater flexibility and tend to perform well on large-scale datasets exceeding fifty thousand images, they may not be as effective in medical domains where the amount of training data is limited and object characteristics are more consistent.

In this context, the performance advantage of YOLOv5 reflects its compatibility with the nature of medical data, as also evidenced by our experimental results. These findings suggest that in specialized domains such as medical imaging, model performance depends more on the suitability of the architecture to the task and dataset characteristics than on the novelty or complexity of the model itself.

**Fig. 9:**
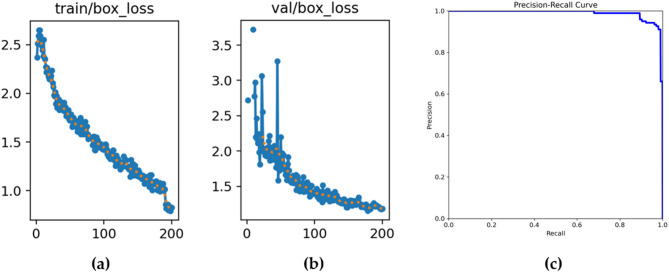
Loss and precision-recall curve graphs (**a**) Training loss curve of bounding box regression, showing a consistent decrease across epochs. (**b**) Validation loss curve of bounding box regression, confirming stable convergence without overfitting. (**c**) Precision–Recall (PR) curve of the trained YOLOv5 model, achieving a high mAP@0.5 of 0.994 for tumor detection.

**Fig. 10:**
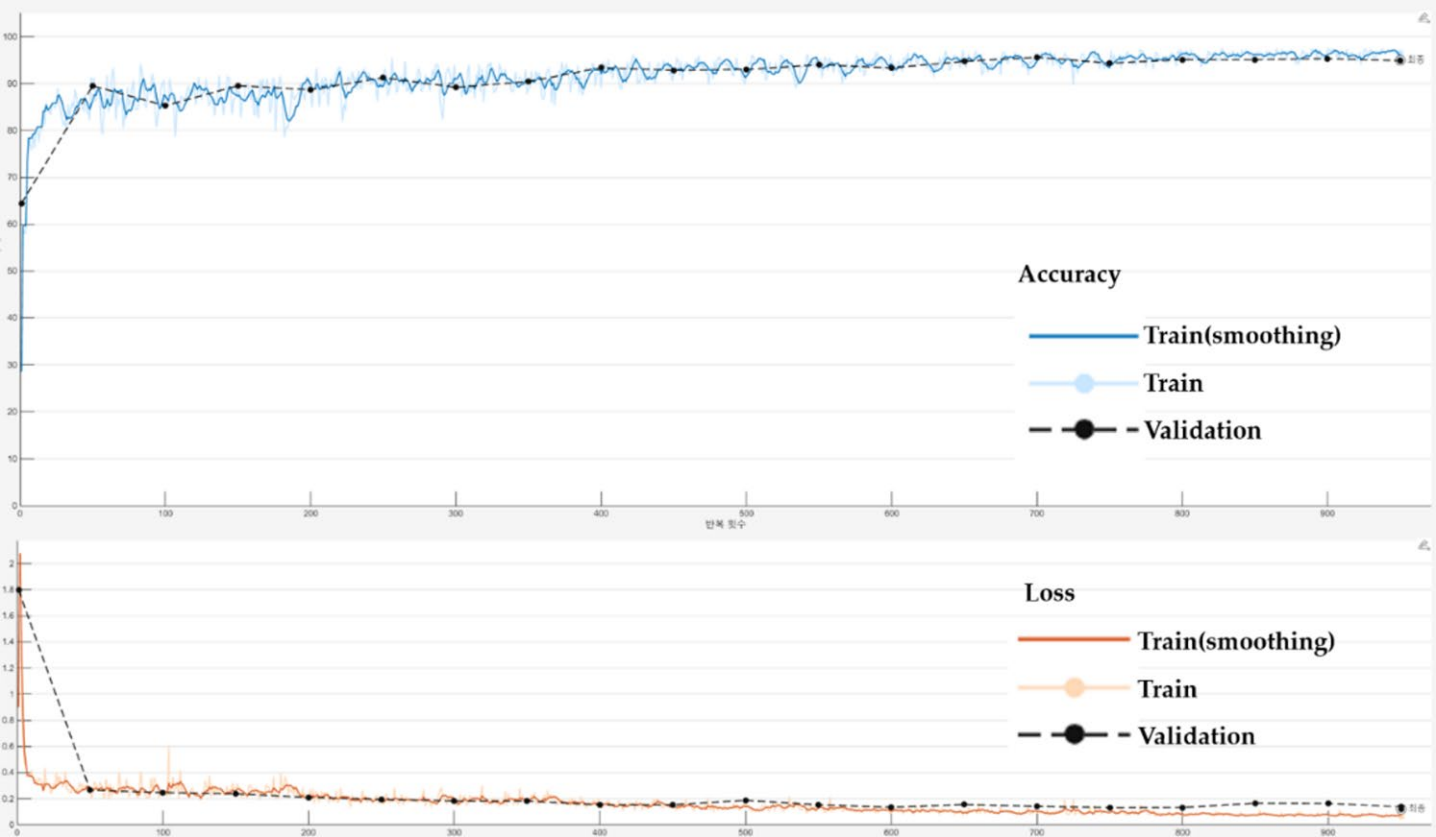
Training and validation curves for the U-Net segmentation model. (Top) Accuracy curves showing consistent improvement in both training and validation sets. (Bottom) Cross-entropy loss curves demonstrating stable convergence without overfitting.

As shown in Fig. 9(a) and (b), both training and validation box loss steadily decreased throughout training, indicating stable optimization. In addition, the PR curve in Fig. 9(c) demonstrates that the model achieved a strong detection performance with mAP@0.5 of 0.994. For the U-Net segmentation model, training was conducted using the Adam optimizer with an initial learning rate of 0.001. A piecewise learning rate schedule was applied, reducing the learning rate by a factor of 0.3 every 10 epochs. The model was trained for 50 epochs with a mini-batch size of 8, and early stopping was triggered if the validation loss did not improve for 4 consecutive validation checks. The training and validation curves shown in Fig. 10 confirm stable convergence without signs of overfitting.

### Performance of BBT segmentation

After the BBT was detected, a padding of 25 pixels was added on all sides of the detection result to prevent any potential loss of contour information. Following this, we proceeded with the segmentation step. Segmentation played a crucial role in our proposed FusionX-BBTNet, as it directly determined the accuracy of the measurements related to the size, area, and shape of the BBT. Rather than simply inputting the original X-ray image into a deep learning model and applying fine-tuning, we generated a new dataset referred to as the FusionX-BBT dataset. This dataset was specifically designed to enhance feature representation by combining the original grayscale image with a wavelet-transformed, three-channel image, resulting in a four-channel input.

For the segmentation model, we adopted U-Net, which is widely used in medical image segmentation tasks due to its effectiveness and reliability. To validate the contribution of the FusionX-BBT dataset, we conducted an ablation study comparing the performance of models trained on the FusionX-BBT dataset with those trained on the raw X-ray images. The details of this comparison are presented in Table 2.

**Table 2:** Performance comparison between the Raw X-ray image dataset and the FusionX-BBT dataset using Unet models.

Model	Dataset	Mean Acc	Mean IOU	BF1 score
Unet	Raw	0.9833	0.9049	0.9759
	FusionX-BBT	0.9844	0.9176	0.9797
	Raw + FusionX-BBT	0.9878	0.9376	0.9827

In this study, cross-entropy loss was used to update the model weights during neural network training. The performance of semantic segmentation was evaluated using mean accuracy, mean Intersection over Union (IoU), and the Boundary F1 Score (BF1 Score). Mean IoU is one of the most commonly used evaluation metrics in semantic segmentation, as it enables the comparison of predicted pixel accuracy against the ground truth. Similarly, the BF1 score quantifies the similarity between the contours of the segmented image and those of the ground truth, focusing on edge-level accuracy. As shown in Table 2, the use of the FusionX-BBT dataset alone, even with a standard U-Net architecture, led to notable improvements in all three metrics when compared to the raw X-ray dataset. Specifically, the model achieved a mean accuracy of 0.9844, a mean IoU of 0.9176, and a BF1 score of 0.9797. These results indicate that the new dataset had a substantial positive impact on the segmentation performance. Furthermore, when the FusionX-BBT dataset was concatenated with the original grayscale data to form a four-channel input, performance improved even further. In this setting, the model achieved a mean accuracy of 0.9878, a mean IoU of 0.9376, and a BF1 score of 0.9827, representing the best overall results. Although the fusion method may appear structurally simple, it was purposefully selected to ensure interpretability, computational efficiency, and real-world applicability. This result supports the clinical value of our strategy even in the absence of complex attention-based fusion. Nonetheless, more advanced fusion techniques such as attention-based or frequency-domain methods will be actively considered in future work to further enhance model performance.

To illustrate the training behavior of the U-Net model, Fig. 10 shows the accuracy and loss curves for both training and validation sets. The curves demonstrate stable optimization, with no sign of overfitting throughout the training process.

### Result using public dataset BTXRD

**Fig. 11:**
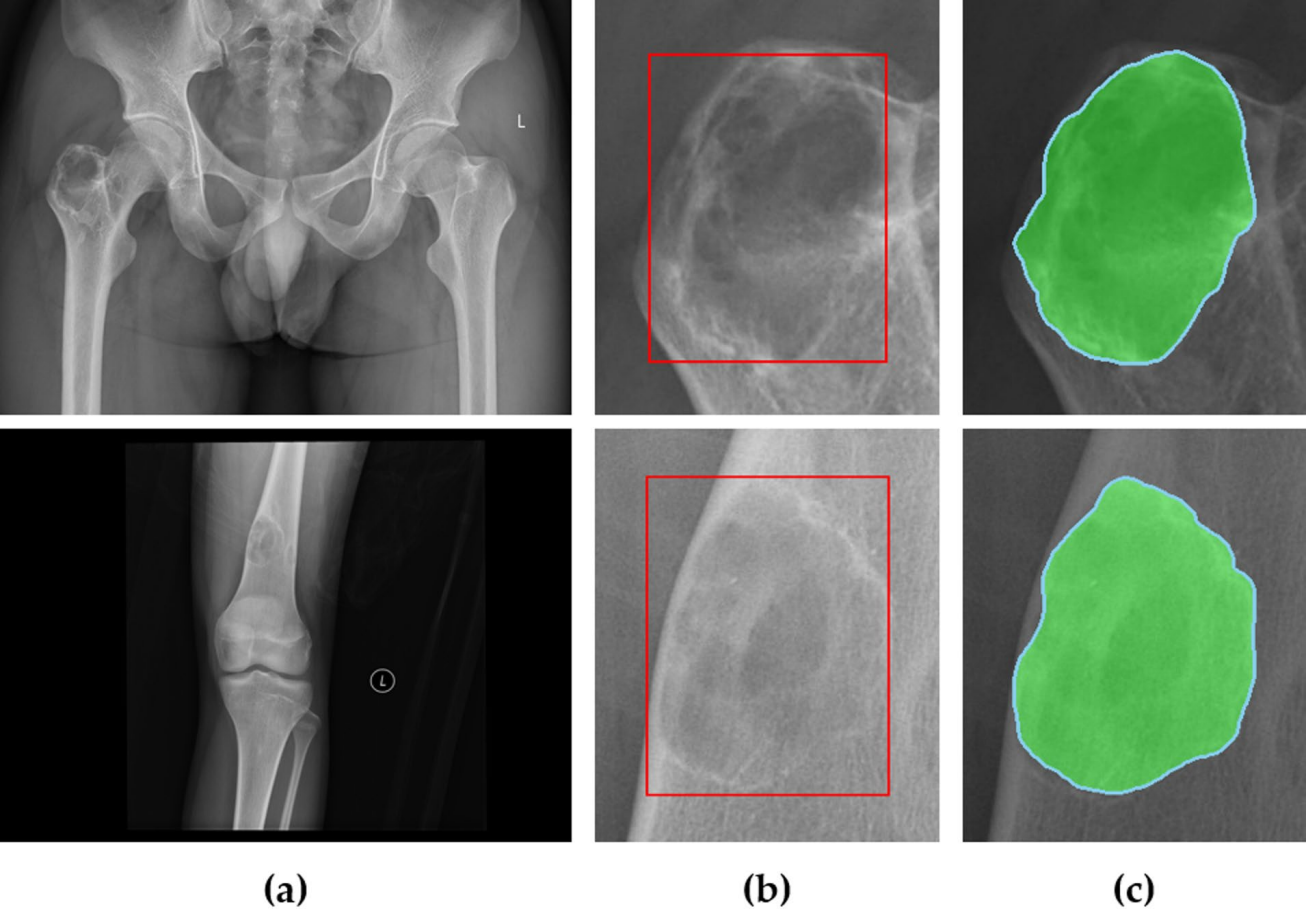
Example of benign bone tumor (BBT) analysis using BTRXD images (**a**) Original BTRXD images (**b**) Detection results (red bounding boxes) (**c**) Segmentation results with light green overlay and cyan edge.

To evaluate the generalizability of the proposed BBT detection and segmentation framework, we tested our method on the publicly available BTRXD dataset^33^, which includes X-ray images of bone tumors acquired under various conditions. Without retraining, the model achieved a mean Average Precision (mAP@0.5) of 0.974 for detection and a mean IoU of 0.9258 for segmentation on a subset of 200 images, demonstrating strong robustness across domains. As illustrated in Fig. 11, the framework accurately localized and segmented benign bone tumors even in external images. However, since the BTRXD dataset does not include a scale bar, it was not possible to measure tumor size in real-world units, which remains a limitation of this evaluation.

### Result of detecting edge of BBT and scale bar

Accurate segmentation using the FusionX-BBT dataset enables reliable edge detection by utilizing the resulting label information. Based on the formulation described in Sect. 2.2.4, we extracted the edge for each dataset in a consistent and computationally precise manner. As illustrated in the corresponding figure, the edges derived from the segmentation results were used for further analysis, including the comparison of tumor size, area, and shape over time.

**Fig. 12:**
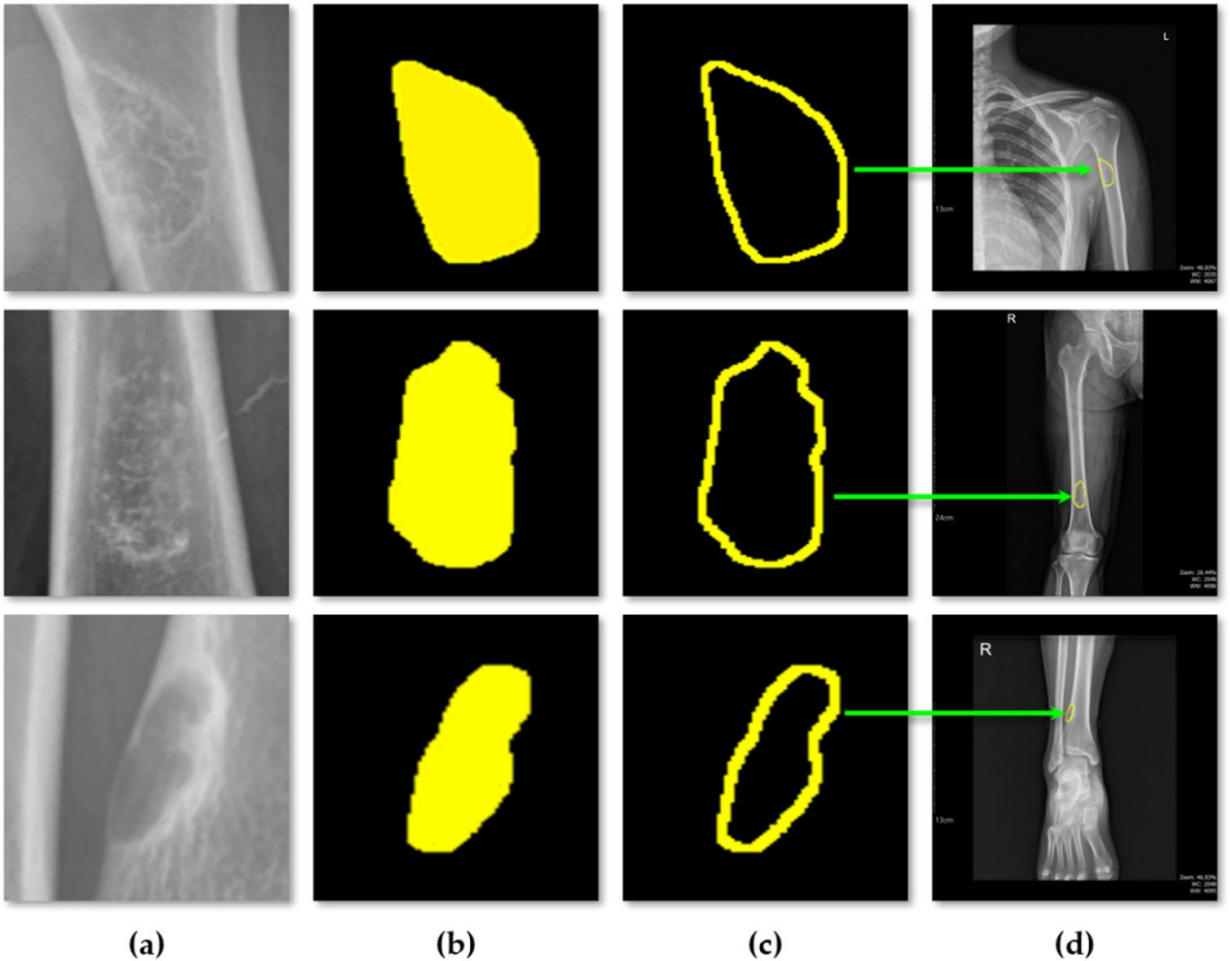
(**a**) BBT image cropped by BBT detection (**b**) BBT segmentation result (**c**) Edge detection was applied to (**b**) and the line width was increased for better visualization. (**d**) Edge detection results overlayed to original X-ray image.

To accurately measure the actual length of the BBT, it was necessary to determine the real-world distance corresponding to each pixel in the X-ray image. Fortunately, all X-ray images included a scale bar that could be used for this purpose. As shown in Fig. 12, we successfully extracted the scale bar and measured its height in pixels. In addition, we applied EasyOCR to automatically recognize the labeled length on the scale bar and determine the corresponding real-world distance in centimeters.

**Table 3:** Quantitative comparison of the extracted scale bar and OCR results with the ground truth.

Data	Patient #1	Patient #2	Patient #3
Result of scale bar and OCR			
**Pixel of scale bar (px)**	423	451	431
**Length of scale bar (cm)**	13	24	13
**Distance per pixel (mm)**	3.25	1.75	3.31
**Real distance**	3.25	1.75	3.30
**Deviation**	0	0	0.01

As shown in Table 3, the length of the scale bar varied across different datasets. Nevertheless, it was accurately detected in all cases, and the OCR system reliably extracted the labeled distance values. Based on this information, we calculated the pixel-to-millimeter conversion ratio for each image. The converted values were then compared to the ground-truth measurements obtained by clinical experts, and the results demonstrated a high degree of consistency between the two, confirming the validity of our approach.

### Result of calculation of BBT size and area

**Fig. 13:**
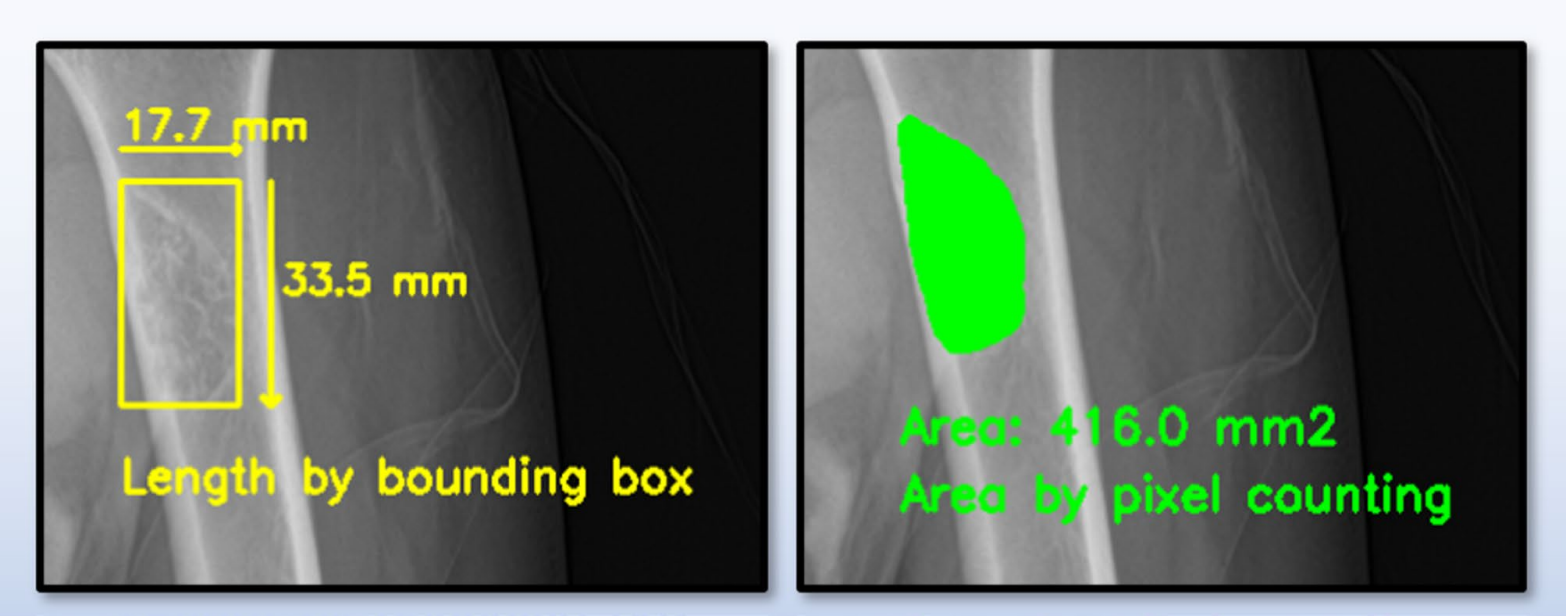
Approach for measuring the size and area of BBT.

**Fig. 14:**
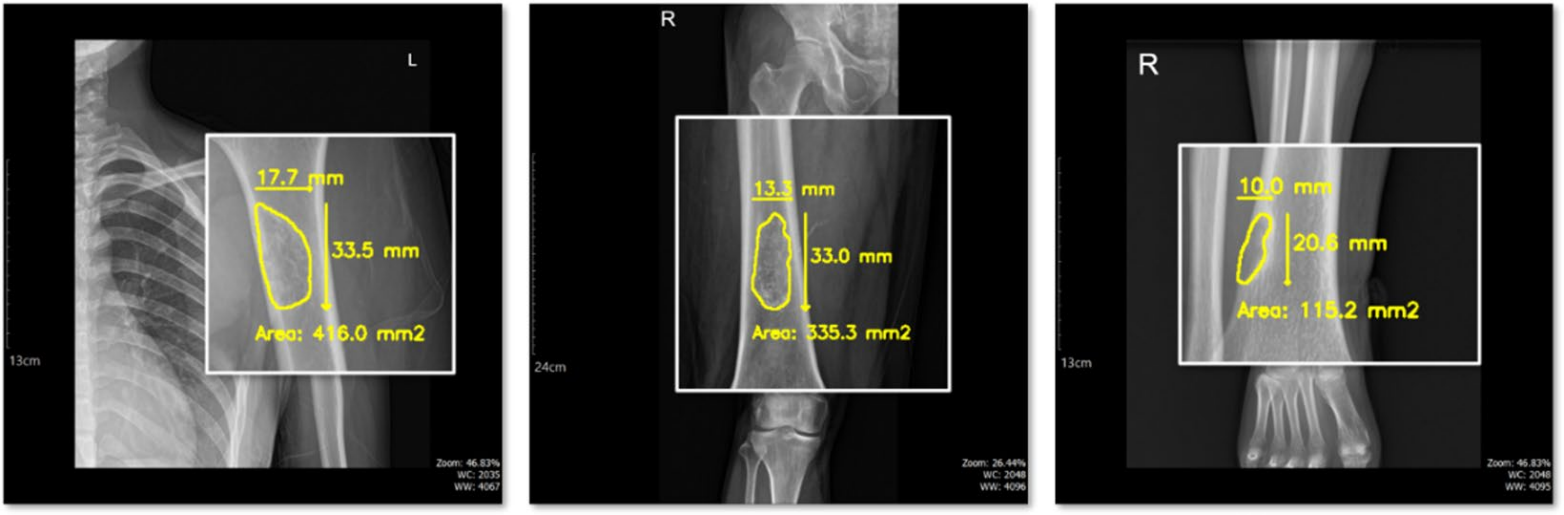
Results of BBT size and area measurement.

To quantitatively measure the size and area of the BBT, we first performed detection and segmentation to accurately localize the tumor and delineate its contour. Subsequently, the scale bar within each X-ray image was identified using EasyOCR, which allowed us to compute the pixel-to-millimeter conversion ratio. Based on this information, as illustrated in Fig. 13, the size of the BBT was calculated by measuring the width and height of the minimum bounding box enclosing the tumor. The area was computed by counting the total number of pixels within the segmented mask and applying the corresponding conversion ratio to obtain the real-world area in square millimeters as illustrated in Fig. 14. By combining the bounding-box-based approach for linear measurements with the mask-based method for area estimation, we were able to evaluate the physical size of the BBT with high precision.

**Table 4:** Results of BBT size, area measurement, ground truth and the deviation include percentage.

Test data number	Predicted			Ground Truth		Deviation	
	Width (mm)	Height (mm)	Area (mm^2^)	Width (mm)	Height (mm)	Width (mm)	Height (mm)
1	17.70	33.50	416	17.89	33.58	0.19	0.08
2	13.30	33.00	335.3	13.40	32.92	0.10	0.08
3	10.00	20.60	115.2	10.09	20.40	0.09	0.20
4	7.04	18.96	66.38	7.01	18.88	0.03	0.08
5	6.12	12.85	54.97	6.08	12.81	0.04	0.04
6	24.78	41.91	739.81	24.76	41.98	0.02	0.07
7	7.34	13.46	71.3	7.41	13.55	0.07	0.09
8	11.62	20.49	130.24	11.68	20.61	0.06	0.12
9	5.51	15.60	47.3	5.51	15.58	0	0.02
10	11.62	16.21	135.39	11.57	16.20	0.05	0.01

Since it was not feasible to include all test data in the manuscript, we selected 10 representative cases and presented them in Table 4. Among these, the largest deviation was observed in the width measurement of 1 st data, with a percentage deviation of 1.06%. The smallest deviation occurred in the width measurement of 9th data, where the predicted value exactly matched the ground truth with a deviation of 0%. Across the 10 test cases, the average deviation in width was 0.58%, the average deviation in height was 0.38%, and the overall average deviation was 0.46%. These levels of deviation are considered sufficiently accurate for practical clinical use.

Regarding area measurement, the current clinical practice generally estimates lesion area by multiplying the width and height. Our research team recognized that such measurements are typically used as reference indicators during longitudinal follow-up, rather than being treated as absolute values. Moreover, we identified a limitation of this method: it may not accurately reflect area changes when the lesion exhibits irregular or asymmetric shapes. In contrast, the method proposed in this study calculates lesion area directly by counting the number of pixels within the segmented bone lesion region on X-ray images. Therefore, the proposed approach is fundamentally different from the conventional width × height estimation method and is not directly comparable.

Future research will aim to validate the clinical significance of the pixel-based area measurement approach and develop algorithms to correct for rotation variability during X-ray acquisition. Further details on these plans are discussed in the Discussion section.

### Result of sequential BBT dataset comparation

Using the measurements obtained through the aforementioned process, we proceeded to the final objective of this study, which is the analysis of time-sequential data. For a single patient, X-ray images acquired at intervals ranging from six months to one year were analyzed. The area and shape of the BBT were computed for each time point and visualized together in a single composite image to facilitate longitudinal comparison.

**Fig. 15:**
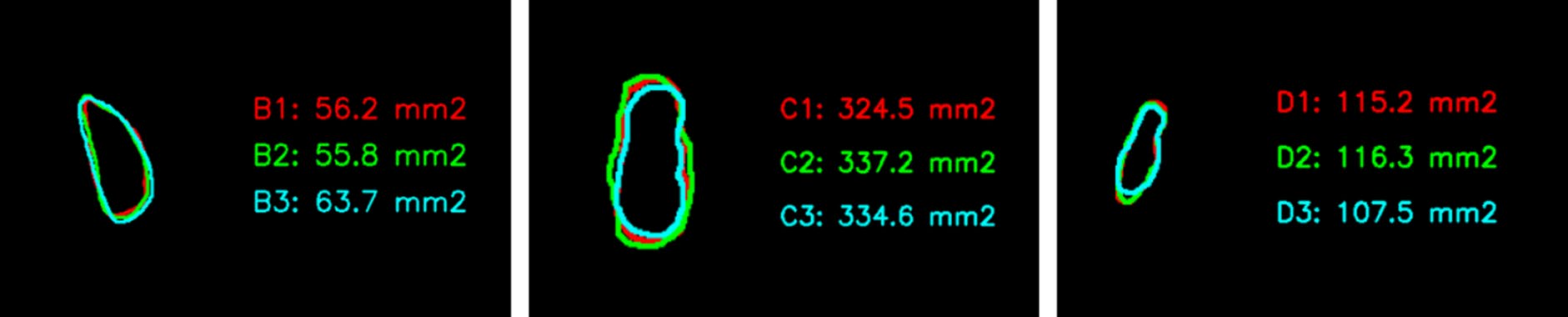
Results of sequential BBT dataset comparation.

Figure 15 visually presents the temporal changes in the area and shape of the BBT in a single patient across multiple time points. Contours extracted from X-ray images taken at intervals of six months to one year were overlaid using different colors (red, green, and blue), with all contours aligned to a common reference point. This alignment minimizes the influence of positional differences and imaging angles, thereby allowing a more accurate assessment of lesion-specific changes. The visual comparison of BBT shapes enables experts to evaluate whether the tumor contours remain stable or become irregular over time. Such shape variations may serve as important supplementary information for estimating disease progression or inferring histopathological characteristics. In particular, morphological changes can be indicative of potential malignancy, making contour comparison a meaningful tool to support the qualitative judgment of clinical experts.

In addition to shape analysis, the automatic calculation of BBT area provides a reliable quantitative indicator. Tumor size is often estimated subjectively based on the clinician’s experience, and manual measurement can be time-consuming and prone to inter- and intra-observer variability. In this study, we used the pixel-to-millimeter conversion factor obtained from the scale bar to calculate the actual BBT area in square millimeters. This approach produced objective and reproducible data, which can support diagnostic decisions and longitudinal monitoring in clinical practice.

In conclusion, this visualization serves not only as a tool for reviewing medical images, but also as a comprehensive aid that combines qualitative interpretation of shape changes with quantitative assessment of area variation. It has the potential to facilitate faster and more accurate decision-making in the clinical workflow.

## Discussion

In this study, we proposed an automated system for quantitatively analyzing the size and morphological changes of BBTs using X-ray images. Throughout the development of this system, several technical considerations emerged.

First, directly applying general semantic segmentation techniques to X-ray images presents significant challenges. This is primarily due to the extremely small proportion of the image that the BBT occupies, with the majority consisting of background. In such cases, segmentation models are prone to misclassifying the tumor as background or failing to accurately capture its contours. To address this issue, we adopted a two-step approach. The tumor was first localized using an object detection model. To prevent contour loss, a fixed padding was applied to the detected region before performing precise segmentation. This two-step strategy proved effective for identifying small lesions and holds strong potential for applications in high-resolution medical imaging.

Second, the proposed FusionX-BBT dataset contributed to improving segmentation performance by overcoming the limitations of conventional grayscale X-ray images. Medical images frequently suffer from noise and low contrast, which can obscure lesion boundaries. To mitigate this, wavelet transform was applied to extract three components—LL, LH, and HL—which were then recombined into a new RGB image. This representation provided sharper and more structurally distinct input features. In particular, the LH and HL components emphasized edge information in horizontal and vertical directions, respectively, helping the model better capture tumor contours. This enhancement was reflected in the improved Boundary F1 Score, a metric that directly measures the precision of contour segmentation and is closely related to the study’s goal of shape-based comparison. In addition, the input structure was designed to enhance feature representation by combining RGB-mapped wavelet components (LL, LH, HL) with the original grayscale image, forming a four-channel input. This allowed the model to simultaneously capture spatial structure, edge details, and intensity information. While the overall framework adopts a conventional YOLO-U-Net combination, this specific multi-channel fusion strategy has been rarely explored and contributed meaningfully to the improved segmentation performance observed in this study.

Third, time-sequential analysis of BBT area and shape plays a critical role in determining whether the lesion has progressed. Traditionally, clinicians have had to manually compare X-ray images acquired at different time points, a process that is not only labor-intensive but also subject to individual interpretation. The automated measurement and alignment system proposed in this study allows BBT regions from different time points to be overlaid using a common centroid, enabling intuitive assessment of changes in both shape and area. The automated area calculation, in particular, provides objective and reproducible data that can support reliable long-term monitoring and treatment evaluation. This system has the potential to compensate for the shortage of radiology specialists while significantly improving the diagnostic efficiency of general clinicians.

Despite its strengths, our approach has several limitations. The BBT dataset used in this study was collected exclusively from a single CSU, and thus lacked diversity and scale. As a result, we were unable to extensively test a wide range of segmentation architectures. With access to a larger and more varied dataset, future studies could evaluate more advanced models, such as TransUNet or Swin-Unet, allowing for more systematic comparisons. In addition, most of the BBTs included in this study were associated with specific diagnostic codes and were often localized to particular anatomical regions (such as the humerus or femur), raising the possibility of model overfitting to these regions. This may limit the generalizability of the model to tumors located in other anatomical areas or with different shapes.

To address these limitations, future research will focus on building a more comprehensive BBT dataset through multi-institutional collaboration, incorporating a wider range of anatomical locations and morphological characteristics. We also plan to train deeper network architectures beyond U-Net to systematically evaluate the scalability and performance of our model.

In addition, future studies will aim to enhance the area measurement methodology. While conventional clinical practice estimates lesion area by simply multiplying the width and height, our study proposes a pixel-based direct area calculation method using segmentation outputs. This new approach allows for a more accurate and objective assessment of lesion changes over time, particularly for irregularly shaped lesions. Furthermore, considering that variations in patient positioning and bone rotation during X-ray acquisition can significantly affect lesion appearance, we plan to develop preprocessing algorithms to correct for rotational discrepancies. Such corrections are expected to improve the precision of longitudinal shape comparisons and area measurements.

## Conclusion

Although X-ray remains the most fundamental and widely used imaging modality in the diagnosis of bone tumors, quantitative comparison between images and analysis of morphological changes are still largely performed manually. In the case of BBTs, temporal evaluation of size and shape changes can provide valuable diagnostic information. However, tools that enable objective quantification of such changes are currently lacking.

To address this issue, we proposed FusionX-BBTNet, a novel framework that enables automated localization, contour extraction, size measurement, and shape analysis of BBTs from X-ray images, along with time-sequential comparison. The system leverages YOLO-based object detection to effectively identify small lesions and employs a wavelet-transformed FusionX-BBT dataset to enhance segmentation performance. Using this approach, the U-Net model trained on the combined Raw + FusionX-BBT dataset achieved a Mean Accuracy of 0.9878, a Mean IoU of 0.9376, and a Boundary F1 Score of 0.9827, showing notable improvements over models trained on Raw images alone.

In terms of size measurement, the proposed method demonstrated high accuracy. Among 10 representative test cases, the average deviation in width measurement was 0.58%, in height measurement was 0.38%, and the overall average deviation across all measurements was 0.46%. These results indicate that the proposed system can provide clinically acceptable precision for assessing tumor size changes over time.

FusionX-BBTNet has the potential to serve as a valuable clinical tool, assisting radiologists and clinicians by complementing visual interpretation and contributing to more accurate diagnosis and treatment planning. In particular, the framework may be especially useful in settings where radiology expertise is limited or in clinical scenarios requiring long-term monitoring of tumor progression.

Future work will focus on expanding the dataset to include a wider range of anatomical sites and a more diverse patient population. Additionally, comparative studies with transformer-based segmentation models such as TransUNet and Swin-Unet will be conducted to further evaluate performance. Furthermore, efforts will be made to integrate the system with existing PACS infrastructure, enabling real-time application within the clinical workflow as part of a comprehensive clinical decision support system.

## Data Availability

The datasets generated and/or analyzed during the current study are not publicly available due to restrictions imposed by the Institutional Review Board (IRB) of Chosun University Hospital, but are available from the corresponding author on reasonable request.
